# Validation of a Low-Cost Whole Slide Imaging System for Pathological Diagnosis of Gastric Ulcers in Biopsy Specimens by Pathology Residents

**DOI:** 10.31557/APJCP.2026.27.1.319

**Published:** 2026-01-21

**Authors:** Warut Thinpanja, Worakit Kaewnopparat, Chatdhee Stithsuksanoh, Pornchai Sooksaen, Phirasit Chaijitrawan, Chetiyaphon Khueankaeo, Natnalin Chumponpanich, Treepob Tassanawarawat, Natcha Poungmeechai, Sompon Apornvirat, Thiyaphat Laohawetwanit

**Affiliations:** 1 *Division of Pathology, Thammasat University Hospital, Pathum Thani, Thailand.*; 2 *Division of Pathology, Chulabhorn International College of Medicine, Thammasat University, Pathum Thani, Thailand.*

**Keywords:** Gastric ulcer, whole slide imaging, validation, diagnosis, pathology residents

## Abstract

**Objective:**

This study aimed to validate a low-cost whole slide imaging (WSI) system, the MoticEasyScan Pro 6 (Motic, Hong Kong), combined with consumer-grade laptops, for the evaluation of gastric ulcer biopsies by pathology residents.

**Methods:**

Sixty-six gastric biopsy slides were scanned at 40× magnification and reviewed by nine pathology residents across three training levels. Each resident interpreted both digital and glass slides for malignancy, *Helicobacter pylori* (*H. pylori*), and intestinal metaplasia (IM) subtypes, with a one-month washout period between formats. Diagnostic agreement was assessed using percentage agreement and kappa statistics, while paired t-tests were used to compare diagnostic times.

**Results:**

Diagnostic agreement between digital and glass slides was highest for malignancy (93.8%, almost perfect), followed by IM (82.6%, substantial) and *H. pylori* (67.8%, fair). Agreement for incomplete IM was significantly lower than for complete IM (70.6% vs. 82.4%, p = 0.02). Discordant diagnoses most frequently involved mild *H. pylori* infection and incomplete IM. Six of nine residents required more time to evaluate digital slides compared to glass slides (138 vs. 90 seconds, p < 0.01), though diagnostic accuracy and time taken were not correlated with training level. Factors contributing to low diagnostic agreement included subtle histologic features, misinterpretation of pseudogoblet cells, overlooked small foci of IM, and inconspicuous microorganisms.

**Conclusion:**

Low-cost WSI systems are feasible for resident training in gastric ulcer biopsy interpretation, especially for distinguishing malignancy. However, lower agreement for *H. pylori* and incomplete IM highlights the challenges of recognizing subtle histologic features on digital slides. Incorporating structured digital pathology training and increasing exposure to WSI during residency may improve diagnostic performance.

## Introduction

Gastric ulcers can be either benign or malignant, and their histopathologic evaluation is essential for guiding patient care. Accurate interpretation is critical, as failure to detect malignancy may delay treatment and worsen prognosis, whereas overdiagnosis can lead to unnecessary surgical intervention. Diagnosing these lesions can be challenging, particularly when clinical information is limited at the time of biopsy [1]. This difficulty is further compounded in settings where endoscopists have limited experience or when the quality of endoscopic equipment is suboptimal [2]. Certain histologic features, such as *Helicobacter pylori* (*H. pylori*) infection and intestinal metaplasia (IM), especially the incomplete subtype, have important clinical implications that can influence management decisions and surveillance strategies.

The use of whole slide imaging (WSI) for primary diagnosis has expanded rapidly following the COVID-19 pandemic [3]. Several digital slide scanners have been approved by regulatory bodies including the United States Food and Drug Administration for primary diagnosis. As digital pathology becomes increasingly integrated into routine diagnostic practice and examination processes [4], early exposure during residency is essential to ensure diagnostic competence. Despite this growth, pathology residents in many resource-constrained institutions still have limited access to WSI systems during training. This limited exposure may hinder development of diagnostic skills using digital slides, which are increasingly used in education, consultation, and practice.

Previous validation studies of WSI in gastrointestinal pathology have primarily focused on experienced pathologists using high-cost systems [5, 6]. Evidence supporting the use of more affordable alternatives for resident training remains limited [7]. In particular, few studies have assessed the ability of less experienced trainees to identify diagnostically subtle but clinically important features [8]. This study aims to validate a low-cost WSI system for evaluating gastric ulcer biopsies by pathology residents.

## Materials and Methods

### Case Retrieval

All consecutive cases labeled as “gastric ulcer” on the pathology request form and initially diagnosed by a gastrointestinal pathologist (TL) were included. Non-neoplastic biopsy slides were stained with Giemsa to enhance the detection of *H. pylori*. The extent of *H. pylori* infection and intestinal metaplasia was assessed using visual analogue scales as outlined in the updated Sydney System for the classification of gastritis [9]. For each case, one representative slide was selected. Key histologic features malignancy, *H. pylori*, IM, complete IM, and incomplete IM were independently assessed by another pathologist (SA) to establish the reference standard. In cases of diagnostic disagreement, both pathologists (TL and SA) reviewed the slides together and reached a consensus diagnosis.

### Slide Digitalization

Glass slides were scanned with a 40× objective lens on the MoticEasyScan Pro 6 (Motic, Hong Kong) using the high-precision autofocus mode, yielding a resolution of 0.26 µm/pixel. The resulting digital images measured 35,441–88,089 pixels in width and 223,614–258,284 pixels in height. Two pathologists (TL and SA) evaluated the quality of each digital slide. Slides with blurred images were rescanned.

### Participants

After a didactic lecture on interpreting gastric biopsy specimens, nine pathology residents participated in the evaluation of both digital and glass slides, with a one-month washout period between sessions. The group included four first-year residents (6 months of experience), three second-year residents (1.5 years of experience), and two third-year residents (2.5 years of experience). All had experience interpreting gastric biopsies on glass slides in routine practice but minimal exposure to digital slides. For each slide, they first determined whether the ulcer was benign or malignant. If benign, they assessed the presence of *H. pylori* and IM, including classification into complete or incomplete subtypes. The diagnostic time for each digital and glass slide was recorded.

### Data Analysis

Diagnostic agreement for each histologic feature was assessed using percentage agreement and kappa statistics. Kappa values were categorized to reflect levels of agreement: less than 0 indicated poor agreement, 0.01 to 0.20 slight agreement, 0.21 to 0.40 fair agreement, 0.41 to 0.60 moderate agreement, 0.61 to 0.80 substantial agreement, and 0.81 to 0.99 almost perfect agreement. Cases with discordant diagnoses in more than 33% of participants (more than 3 out of 9) were subjected to further analysis. Paired t-tests were used to compare diagnostic times for digital and glass slide interpretation for each participant. A p-value of less than 0.05 was considered statistically significant. All statistical analyses were performed using STATA version 18 (StataCorp., Texas, USA).

## Results

### Specimen Characteristics

A total of 66 specimens, including 18 malignant ulcers and 48 benign ulcers, were retrieved. All of the former were adenocarcinoma. Specimen characteristics are summarized in Table 1. 

### Diagnostic Agreement of Each Histologic Finding

**Figure 1:**
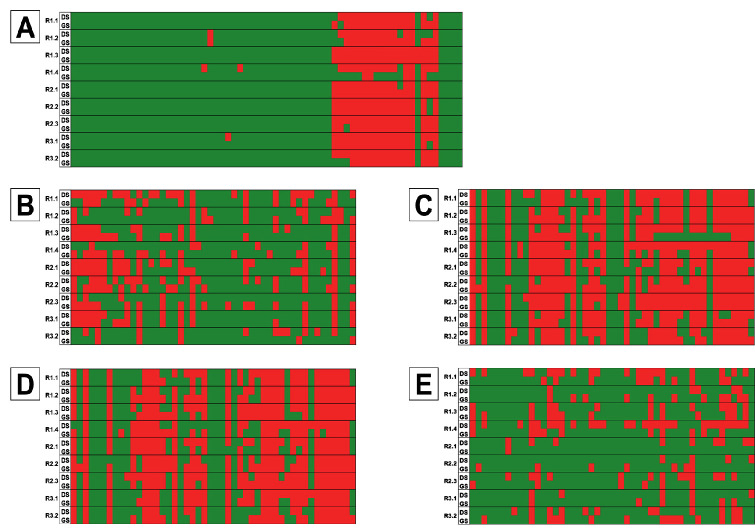
Heat Maps Illustrating Diagnostic Agreement between Digital (DS) and Glass Slides (GS) for Each Participant Across All Cases. Red indicates presence, and green indicates absence of the target finding. Participants are labeled by training level: R1 = 1st-year resident, R2 = 2nd-year resident, R3 = 3rd-year resident. Diagnostic categories include: (A) poorly differentiated adenocarcinoma, (B) *Helicobacter pylori*, (C) intestinal metaplasia, (D) complete intestinal metaplasia, and (E) incomplete intestinal metaplasia.

Heat maps illustrating diagnostic agreement for each histologic finding including adenocarcinoma, *H. pylori*, IM, complete IM, and incomplete IM are shown in Figure 1. Table 2 summarizes the mean percentage agreement, kappa values, and corresponding interpretations comparing digital and glass slide diagnoses, stratified by year of pathology residency. Among all histologic findings, agreement for adenocarcinoma was the highest (% agreement = 93.8), indicating almost perfect agreement. IM and complete IM showed substantial agreement, while *H. pylori* and incomplete IM demonstrated fair agreement. Notably, agreement for incomplete IM was significantly lower than for complete IM (70.6% vs. 82.4%, p = 0.02). First-year residents showed lower levels of agreement across all findings compared with second- and third-year residents.

### Analysis of Histologic Findings of Slides with Low Diagnostic Agreement

**Figure 2:**
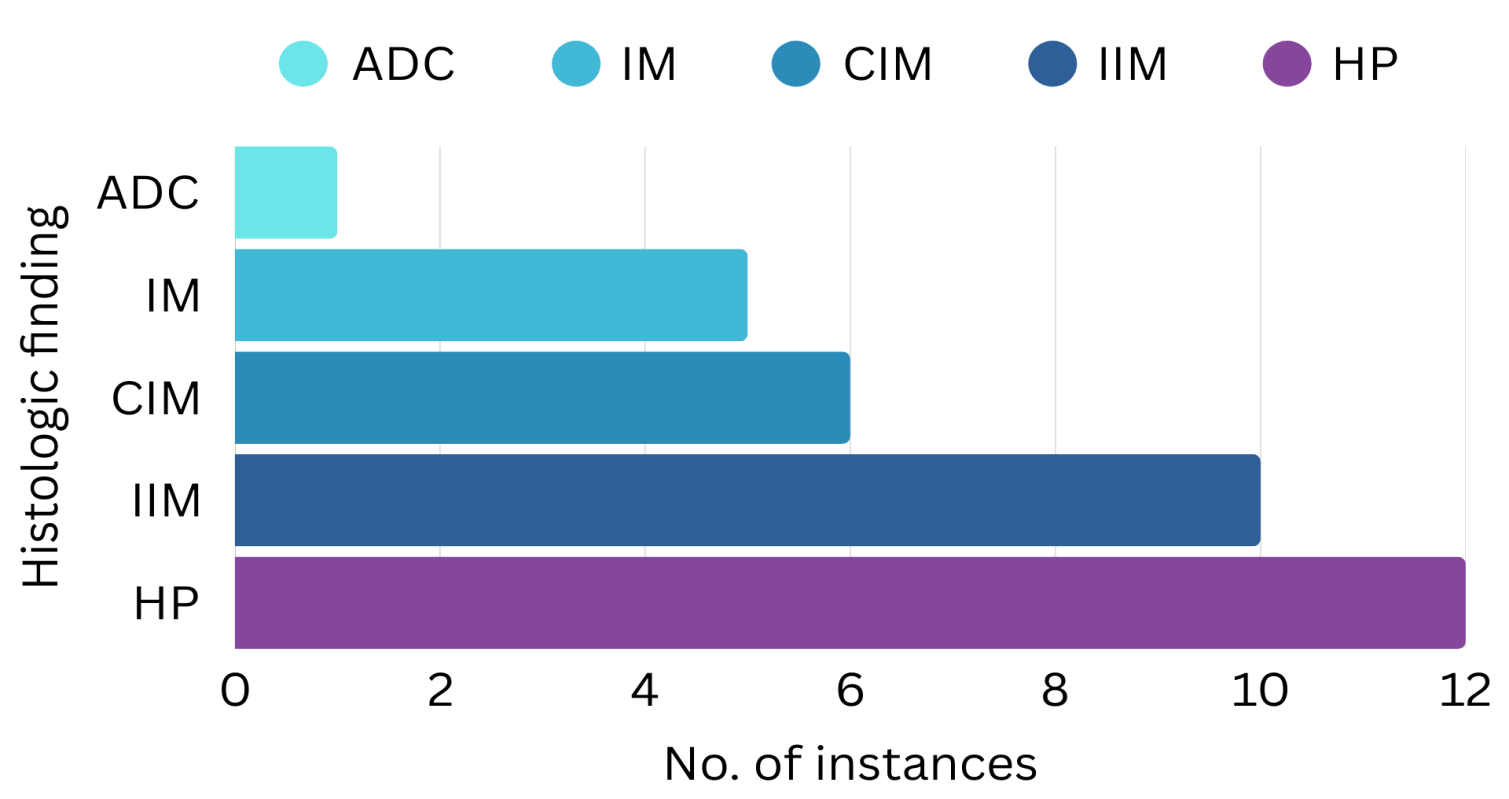
Bar Chart Showing the Number of Histologic Findings in Cases with Low Diagnostic Agreement between Digital and Glass Slides. Abbreviations: ADC, adenocarcinoma; IM, intestinal metaplasia; CIM, complete IM; IIM, incomplete IM; HP, *H. pylori*

A total of 34 instances were identified in which more than one-third of participants (at least four) rendered discordant diagnoses between digital and glass slides (Figure 2). The most common discrepancies involved *H. pylori* (12 instances) and incomplete IM (10 instances). Low diagnostic agreement was typically associated with mild *H. pylori* infection (9/12 instances), IM (4/5 instances), and complete IM (4/6 instances). In contrast, discrepancies in incomplete IM occurred across all degrees of involvement, with mild incomplete IM detected in only 4 of 10 instances.

**Figure 3:**
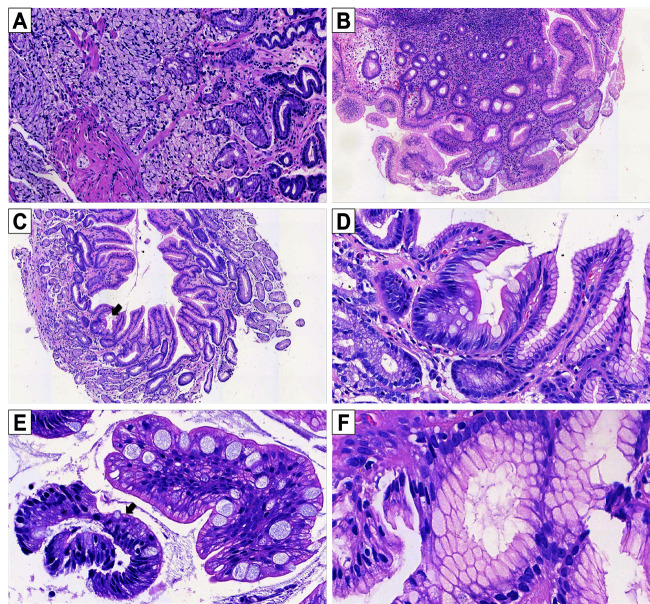
Histologic Findings in Cases with Low Diagnostic Agreement.(A) Poorly differentiated adenocarcinoma. Signet-ring cell carcinoma with clear, vacuolated cytoplasm and minimally pleomorphic nuclei may mimic gastric xanthoma. Infiltration into the muscularis mucosae supports a diagnosis of gastric carcinoma rather than xanthoma.(B) Intestinal metaplasia. Subtle IM is seen adjacent to dilated foveolar mucin-containing cells (pseudogoblet cells) in the lower right corner.(C) Complete intestinal metaplasia, scanning magnification. The subtle focus of complete IM (arrow) is easily overlooked at low magnification.(D) High magnification of panel C showing the same complete IM focus.(E) Incomplete intestinal metaplasia. Incomplete IM with minimal foveolar mucin (arrow) is difficult to recognize.(F) *Helicobacter pylori*. Helicobacter-like organisms are subtly present in a background of mild chronic gastritis.

Review of discordant cases highlighted several factors contributing to interpretive difficulty. Although rare, poorly cohesive carcinoma presented challenges, with signet-ring cell carcinoma occasionally misinterpreted as a non-neoplastic process (Figure 3A). In some cases, dilated foveolar mucin-containing cells (pseudogoblet cells) obscured adjacent areas of IM, complicating recognition (Figure 3B). Small foci of complete IM were frequently overlooked at low magnification (Figure 3C) but became more apparent upon closer inspection (Figure 3D). Incomplete IM with subtle mucinous features also proved difficult to identify (Figure 3E). Similarly, Helicobacter-like organisms were sometimes inconspicuous, particularly in the setting of mild chronic gastritis (Figure 3F).

### Comparison of Time Taken per Slide for Diagnosis

**Figure 4:**
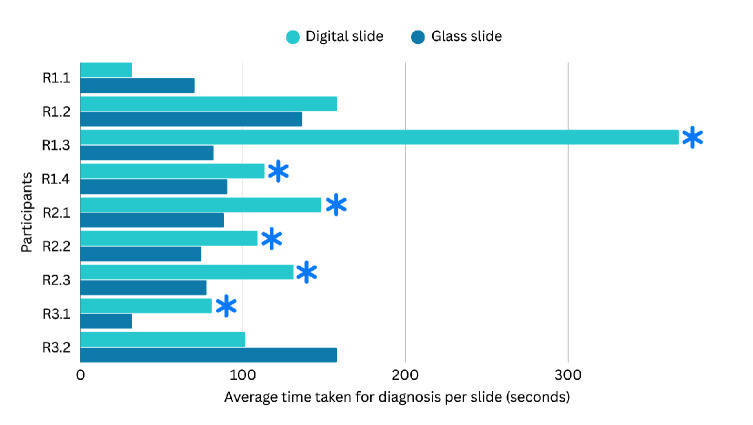
Bar Chart Showing the Average Time Each Participant Took to Diagnose Using Digital Slides and Glass Slides. Participants are labeled by training level: R1 = 1st-year resident, R2 = 2nd-year resident, R3 = 3rd-year resident. Asterisks indicate statistically significant differences between digital slide and glass slide diagnosis times, with all p-values less than 0.01.

The comparison of time taken per slide for diagnosis using digital and glass slides across all participants is illustrated in Figure 4. On average, diagnoses made on digital slides required significantly more time than those made on glass slides (138 vs. 90 seconds, p < 0.01). Six of the nine participants (66.7%) comprising 2 of 4 first-year residents, all 3 second-year residents, and 1 of 2 third-year residents spent more time diagnosing digital slides. In contrast, two residents (1 first-year and 1 third-year) spent more time diagnosing glass slides, although this difference was not statistically significant.

## Discussion

The present study validated a low-cost WSI system for diagnosing gastric ulcer biopsies by pathology residents and demonstrated high diagnostic agreement between digital and glass slides for adenocarcinoma (93.8%, almost perfect). Agreement was moderate to substantial for IM, but only fair for *H. pylori* and incomplete IM. Senior residents generally achieved higher diagnostic agreement than junior ones. Interpretation using digital slides took significantly longer than with glass slides. Common sources of discrepancy included subtle histologic features, particularly incomplete IM and *H. pylori*. These findings support the feasibility of low-cost WSI for resident training and imply the need for greater exposure to digital pathology to enhance recognition of diagnostically subtle features.

Our study followed key recommendations outlined in the College of American Pathologists (CAP) guideline statements [10]. More than 60 cases were included for one use case, specifically the H&E interpretation of gastric ulcer biopsies. Diagnostic concordance between digital and glass slides was assessed by the same observer with a washout period of at least two weeks. Although the overall concordance between digital and glass slide diagnoses was below the 95% threshold, which typically requires further investigation and corrective measures, this percentage should be interpreted with caution due to the limited number of slides used in this study (N = 64). While agreement was almost perfect for adenocarcinoma and substantial for IM, the causes of discordance in the diagnosis of *H. pylori* and incomplete IM, both of which showed only fair agreement, should be carefully examined. Residents should receive training in digital slide interpretation during residency, and early career pathologists are encouraged to validate their performance before using digital slides for primary diagnosis.

Although adenocarcinoma demonstrated almost perfect diagnostic agreement between digital and glass slides among all participants, one case showed discordant diagnoses in more than one third of participants. In this instance, the tumor cells had mildly pleomorphic nuclei and clear, vacuolated cytoplasm, resembling histiocytes seen in gastric xanthoma. This highlights a known pitfall in digital pathology, where subtle nuclear features such as chromatin texture or relative hyperchromasia may be less distinct, potentially leading to diagnostic challenges, particularly when assessing dysplasia [11]. It is important to recognize that in malignant gastric ulcers, the absence of evidence should not be interpreted as evidence of absence. Gastric cancer develops in approximately 2 to 3 percent of patients with gastric ulcers, especially those infected with *H. pylori*. Biopsies obtained from both the base and edges of ulcers during a second or subsequent endoscopy have been shown to enhance early and accurate cancer detection compared to sampling from the edges alone [12]. Regular follow-up endoscopies after ulcer treatment are also associated with higher detection rates of early gastric cancer [13].

The overall agreement for *H. pylori* detection was the lowest among all histologic findings, despite scanning slides at 40× magnification. This may reflect participants’ limited experience in identifying microorganisms on digital slides. Most validation studies to date have used 20× magnification, which reduces scanning time and storage requirements [14]. However, *H. pylori*, Candida albicans, and Giardia duodenalis are microorganisms that are particularly difficult to detect at this resolution. Scanning at 40× magnification provides better visual detail and can increase diagnostic confidence [15]. Even when using immunohistochemistry, identifying *H. pylori* remains challenging under 20× magnification [16]. To address this limitation, pathologists should be advised that higher magnification is essential for reliable detection of microorganisms in WSI. In addition, recognition of characteristic histologic features of *H. pylori* gastritis such as diffuse or nodular lymphocytic inflammation and neutrophilic infiltrates is crucial to improve diagnostic accuracy (i.e., actively searching for *H. pylori*). In equivocal cases, *H. pylori* immunostaining should be employed. In diagnostic practice, several semi-quantitative scoring systems are available for grading the density of *H. pylori*. However, these systems lack clinical significance and show poor inter-observer reproducibility. In routine settings, distinguishing simply between *H. pylori*–positive and *H. pylori*–negative status is considered sufficient [17]. Importantly, the absence of *H. pylori* on histologic evaluation does not exclude the diagnosis of *H. pylori* gastritis, as ancillary tests such as the rapid urease test or other noninvasive methods provide high diagnostic sensitivity [18].

Recent guidelines suggest that patient management, particularly endoscopic surveillance, may differ for individuals with incomplete IM due to a higher risk of progression to gastric cancer compared to those with complete IM [19, 20]. In our study, substantial agreement was achieved for the detection of overall IM and complete IM, but the agreement for incomplete IM was only fair, with low diagnostic concordance regardless of its extent. This suggests that pathology residents experienced difficulty in recognizing incomplete IM. Our findings align with a previous report showing that, even after training, general pathologists and pathology residents demonstrated lower accuracy in identifying incomplete IM compared with IM and complete IM [21]. Unlike gastrointestinal pathologists, who demonstrate high interobserver agreement in IM subtyping [22], non-experts may benefit from additional training to enhance their diagnostic accuracy for incomplete IM. Ancillary stains such as Alcian blue at pH 2.5, which highlights acidic mucins, can aid in distinguishing complete from incomplete IM. In complete IM, goblet cells contain predominantly sialomucins, whereas incomplete IM shows a mixture of sialomucins and sulfomucins that are more readily demonstrated with this stain. Thus, Alcian blue at pH 2.5 remains a practical tool for enhancing recognition and subtyping of incomplete IM.

Findings from this study indicate that six of nine participants required more time to review digital slides compared with glass slides, a finding consistent with previous validation studies [23, 24]. However, this should not be interpreted as evidence that digital slides are inferior. Longer review times largely reflect the adjustment period needed for pathologists to become proficient with (WSI). This “learning effect” is well recognized; after sufficient exposure approximately 500 digital cases the difference in reading time between digital and glass slides becomes negligible [25]. Although using lower scanner magnification can shorten review time, important subtle features or microorganisms may be overlooked [26, 27]. To address these challenges, strategies such as structured training with mandatory case sets, adjusting scanner magnification to suit individual cases, and standardizing laboratory infrastructure and WSI protocols are recommended [5].

There are several limitations in this study. First, the sample size was relatively small, comprising only 66 cases, which may limit the generalizability of the findings. Second, participants were asked to interpret H&E-stained slides and render definitive diagnoses without access to ancillary stains or recuts, which does not fully reflect routine clinical practice where additional tools, such as cytokeratin or *H. pylori* immunostains, are often employed. Third, all malignant cases were adenocarcinomas, preventing evaluation of the WSI system’s performance across other gastric malignancies; however, adenocarcinoma remains the most common subtype encountered in gastric biopsies. Fourth, the number of participants was limited and all were affiliated with a single institution, which may not capture the variability in training environments or represent broader trainee populations. Lastly, the study relied on a single digital slide scanner and consumer-grade laptops, which may restrict the applicability of the results to other hardware or software settings.

In conclusion, this study supports the feasibility of using a low-cost WSI system for resident training in gastric ulcer biopsy diagnosis, especially for malignancy. Lower agreement for *H. pylori* and incomplete IM highlights the need for more digital pathology exposure to improve recognition of subtle features. Integrating WSI into residency programs may enhance diagnostic accuracy, particularly in resource-limited settings.

**Table 1:** Specimen Characteristics

Characteristics	N (%)
Malignant	18 (28.3)
Poorly cohesive carcinoma	13 (72.2)
Tubular adenocarcinoma	5 (27.8)
Benign	48 (72.7)
* Helicobacter pylori*	15 (31.3)
Intestinal metaplasia	34 (70.8)
Complete intestinal metaplasia	33 (68.8)
Incomplete intestinal metaplasia	16 (33.3)

**Table 2:** Diagnostic Agreement Metrics by Year of Pathology Residency Across Histologic Findings.

Histologic findings	%Agreement		Kappa		
	Mean	SD	Value	SD	Level of agreement
Adenocarcinoma					
First-year residents	90.2	6.8	0.71	0.24	Substantial
Second-year residents	97.5	0.9	0.93	0.02	Almost perfect
Third-year residents	95.5	2.1	0.88	0.06	Almost perfect
Overall	93.8	5.5	0.82	0.2	Almost perfect
*Helicobacter pylori*					
First-year residents	63.5	8.4	0.2	0.11	Slight
Second-year residents	73.6	7.9	0.4	0.13	Fair
Third-year residents	67.7	13.3	0.25	0.1	Fair
Overall	67.8	9.3	0.28	0.14	Fair
Intestinal metaplasia					
First-year residents	79.7	8.7	0.58	0.16	Moderate
Second-year residents	84	4.3	0.65	0.08	Substantial
Third-year residents	86.5	1.5	0.72	0.02	Substantial
Overall	82.6	6.5	0.64	0.12	Substantial
Complete intestinal metaplasia					
First-year residents	80.7	4.6	0.6	0.1	Moderate
Second-year residents	84	2.4	0.66	0.04	Substantial
Third-year residents	83.3	5.9	0.66	0.13	Substantial
Overall	82.4	4	0.64	0.08	Substantial
Incomplete intestinal metaplasia					
First-year residents	63.5	17.9	0.18	0.19	Slight
Second-year residents	79.9	8.4	0.24	0.25	Fair
Third-year residents	70.8	0	0.27	0.14	Fair
Overall	70.6	14	0.22	0.18	Fair

Abbreviation: SD, Standard deviation.

## Author Contribution Statement

WT: Conceptualization; Data curation; Formal analysis; Investigation; Methodology; Writing – original draft. WK, CS, PS, PC, CK, NC, TT, NP: Data curation; Investigation; Methodology; Writing - review & editing. SA: Conceptualization; Data curation; Formal analysis; Investigation; Methodology; Writing - review & editing. TL: Conceptualization; Data curation; Formal analysis; Investigation; Methodology; Project administration; Resources; Writing – original draft, review & editing
